# Relationship Between Clinical Manifestations of Acute Rheumatic Fever and Mutations in the FMF-Associated *MEFV* Gene Among Turkish Children

**DOI:** 10.3390/children13060764

**Published:** 2026-05-30

**Authors:** Mustafa Dogan, Metin Tan, Emre Tepeli, Ozlem Gul, Dolunay Gurses, Guven Yenmis, Mehmet Dokur

**Affiliations:** 1 Department of Pediatric Cardiology, Faculty of Medicine, Kocaeli University, Kocaeli 41380, Turkey; 2 Department of Pediatrics, Tekirdag Yaşam Hospital, Tekirdag 59100, Turkey; metintan72@yahoo.com; 3 Department of Genetics, Faculty of Medicine, Istanbul Atlas University, Istanbul 34408, Turkey; dremretepeli@gmail.com; 4 Department of Pediatrics, Diyarbakır Children’s Hospital, Diyarbakir 21000, Turkey; drsivasli@hotmail.com; 5 Department of Pediatric Cardiology, Faculty of Medicine, Pamukkale University, Denizli 20070, Turkey; dolunayk@yahoo.com; 6 Department of Medical Biology, Tayfur Ata Sökmen School of Medicine, Hatay Mustafa Kemal University, Hatay 31060, Turkey; guven.yenmis@mku.edu.tr; 7 Department of Emergency Medicine, Faculty of Medicine, Bilecik Şeyh Edebali University, Bilecik 11000, Turkey; mehmet.dokur@bilecik.edu.tr

**Keywords:** acute rheumatic fever, *MEFV* gene, familial mediterranean fever, arthritis, carditis, mutation

## Abstract

**Highlights:**

**What are the main findings?**

- The *MEFV* exon 2 E148Q variant was more frequent in Turkish children with acute rheumatic fever than in healthy controls.
- No significant difference was found between patients and controls in the distribution of *MEFV* exon 10 variants, while E148Q was the most common variant across ARF subgroups.

**What are the implications of the main findings?**

- The E148Q variant may contribute to genetic susceptibility or phenotypic variation in pediatric acute rheumatic fever.
- *MEFV* variations, particularly E148Q, may deserve consideration in the differential evaluation of Turkish children presenting with acute rheumatic fever and overlapping inflammatory features.
- This study suggests that the development of carditis and arthritis in certain patients with acute rheumatic fever may be associated with genetic susceptibility.

**Abstract:**

**Background/Objectives**: In individuals with a genetic predisposition, acute rheumatic fever (ARF) can manifest as arthritis, carditis, chorea, subcutaneous nodules, and erythema marginatum. It occurs after a latent period of 1–3 weeks of untreated upper respiratory tract infections caused by group A beta-hemolytic streptococci. The presence and severity of carditis determine the prognosis for ARF. Carditis manifests as pancarditis, and although all patients have pericarditis, not all experience a pericardial effusion. Patients with severe carditis exhibit pericardial effusion more frequently. The physiopathology of ARF remains unclear, specifically which patients will experience carditis, arthritis, or chorea. However, the Turkish population has fully clarified the physiopathology and clinical features of Familial Mediterranean fever (FMF), a common rheumatic disease. In the Turkish population, the heterozygous positivity rate for the FMF gene mutation is 15–35%. For these reasons, we examined the presence of FMF gene mutations in our patients to determine whether there is a correlation between the clinical course of ARF and the FMF gene mutation. **Methods**: The study included 60 patients with arthritis (*n* = 11), carditis (*n* = 26), or both (*n* = 23), as well as 60 healthy controls. These pediatric patients underwent screening for mutations in exons 2 and 10 of the *MEFV* gene. **Results**: There was no statistically significant difference between the patient and control groups in terms of the incidence of *MEFV* gene mutations in exon 10. However, in patients with ARF, the exon 2 E148Q variant was significantly more common than in the control group. **Conclusions**: This study suggests a relationship between certain clinical manifestations of ARF and *MEFV* gene mutations in children.

## 1. Introduction

Acute rheumatic fever (ARF) impacts the skin, brain, joints, heart, blood vessels, and connective tissue. It usually manifests 1 to 5 weeks after upper respiratory tract infections caused by group A beta-hemolytic streptococci (GAS) strains [1]. Hospitalization for ARF typically serves to confirm the diagnosis and alleviate symptoms. Persistent cardiac valvular damage, characteristic of rheumatic heart disease (RHD), may develop and emerge as the primary cause of morbidity and mortality in individuals with ARF. When ARF and rheumatic carditis happen together, they can cause persistent valvular dysfunction, which can show up as mitral and/or aortic regurgitation or, in the worst cases, stenosis [2]. The incidence of acute ARF, once the predominant cause of acquired heart disease globally, has decreased in developed nations. We can attribute this decline to improved living conditions, early diagnosis, effective penicillin treatment and prevention, thorough patient follow-up, and advanced diagnostic technologies like echocardiography. Despite this decline, ARF remains a major challenge in underdeveloped and developing countries [3]. Familial Mediterranean fever (FMF) is the most frequent hereditary periodic fever, marked by recurrent inflammatory episodes that impact the abdomen, musculoskeletal system, and skin [4]. Moreover, populations of Mediterranean descent, including Arabs, Armenians, non-Ashkenazi Jews, and Turks, commonly inherit FMF in an autosomal recessive manner. FMF is characterized by a series of acute, recurrent, and brief febrile episodes accompanied by pain, interspersed with variable periods of remission. In 1997, researchers identified a gene responsible for FMF. Biallelic pathogenic variants in this gene cause heightened activation of the pyrin inflammasome, leading to inflammation. Mutations in this Mediterranean fever (*MEFV)* gene, located on chromosome 16, cause FMF. This gene encodes pyrin, a protein essential for regulating inflammation [5,6]. People with FMF mutations often have structural abnormalities in the pyrin protein, which reduces its ability to reduce inflammation in neutrophils. This compromises the body’s ability to control inflammatory responses. Dysregulation results in uncontrolled inflammatory responses, such as peritonitis (inflammation of the peritoneum), fever, and pleuritis (inflammation of the pleura), restricted to specific regions, including joints and skin [7]. Alterations in the *MEFV* gene may trigger a cascade of proinflammatory events. These include the release of inflammatory mediators, increased interleukin-1 (IL-1) production, and inhibition of apoptosis. Even minor stimuli may therefore provoke an exaggerated inflammatory response. This dysregulated inflammatory state may present as disorders with early arthritis-like features through both joint-related and systemic mechanisms. Accordingly, several studies have investigated the inflammatory pathways involved in these diseases [8,9]. Compared with the general population, individuals with FMF have a higher frequency of concomitant inflammatory disorders. These include acute post-streptococcal glomerulonephritis, inflammatory bowel disease, Henoch-Schönlein purpura (HSP), systemic lupus erythematosus (SLE), Behçet’s disease (BD), and polyarteritis nodosa (PN) [10,11,12]. This broader inflammatory background suggests that *MEFV* variation may also influence the clinical expression of ARF. Although the pathogenesis of ARF remains incompletely understood, experts believe that dysregulated responses in both the cellular and humoral immune systems play a significant role [1,9,13].

This study sought to determine whether pericardial effusion and carditis in individuals with ARF were associated with changes in *MEFV* exons 2 and 10.

## 2. Materials and Methods

### 2.1. Sampling

This single-center, retrospective clinical study examined a cohort of 60 children aged 6 to 16 years who were admitted to the Pamukkale University Pediatric Cardiology Clinics from 1998 to 2014. Carditis was diagnosed based on clinical findings and echocardiographic evaluation in accordance with the Jones criteria (1992) for Acute Rheumatic Fever [14]. Echocardiographic assessment was performed by experienced pediatric cardiologists using a cardiac ultrasound/echocardiography device (GE Vivid 7 Imaging System,. GE Vingmed Ultrasound, Horten, Norway). Pathological mitral and aortic regurgitation were defined according to standard Doppler echocardiographic criteria, including visualization in at least two views, a pansystolic (mitral) or pandiastolic (aortic) jet, jet length exceeding physiological limits, and high-velocity flow consistent with pathological regurgitation. Subclinical carditis was defined as the presence of pathological valvular regurgitation detected by echocardiography in the absence of an audible murmur on physical examination. Pericardial effusion was identified by the presence of an abnormal fluid collection within the pericardial space on echocardiography. The severity of valvular involvement was graded as mild, moderate, or severe according to standard pediatric echocardiographic criteria, taking into account regurgitant jet length, jet area, vena contracta width, chamber enlargement, and hemodynamic impact. We applied the updated World Health Organization (WHO) criteria for patients diagnosed after 2003. Patients were not included in the study after the first evaluation if they had an unconfirmed diagnosis of ARF, isolated arthralgia without evidence of arthritis, a history of trauma and clinical signs suggestive of trauma, coexisting vasculitic disease with arthritis or bone dysplasia, congenital cardiac anomalies, or other major systemic disorders. Patients and controls with Familial Mediterranean Fever were excluded based on the Tel-Hashomer diagnostic criteria.

The patient cohort comprised children with arthritis (*n* = 11), carditis (*n* = 26), or both manifestations (*n* = 23). The control group consisted of 60 children matched for age and sex (4–16 years). These children had no inflammatory condition, such as trauma or infection, and no significant clinical abnormality. They also had no chronic disease or evidence of heart disease on physical examination, electrocardiography (ECG), or echocardiography (ECHO). Informed consent, both written and verbal, was secured from the parents of all children involved in the study.

We created a unique form for all patients in both the patient and control groups. This form obtained demographic data, encompassing personal and familial history traits, initial age, age at diagnosis, age at symptom onset, place of birth, parental interpersonal relationships, gender, and the presence of additional concomitant diseases. We enquired about the occurrence of FMF and amyloidosis in their close relatives. All participants underwent a comprehensive physical examination. All participants enrolled in the study underwent a standard echocardiographic evaluation. The evaluation comprised 2D ECHO, PW Doppler, CW Doppler, Color Flow Doppler, and M-mode ECHO. A pediatric cardiologist examined the patient in a supine position without sedation. The pediatric cardiologist acquired echocardiograms from standard precordial positions. Each evaluation lasted approximately 15 to 20 min. All participants received an ECG simultaneously. The control group was defined by inclusion criteria requiring sinus rhythm on the ECG. Both groups excluded individuals diagnosed with FMF or identified as FMF carrier relatives according to the Tel-Hashomer [15] and Yalçınkaya criteria [16].

### 2.2. Molecular Analysis

We collected two milliliters of blood samples from participants to screen for mutations in exons 2 and 10 of the *MEFV* gene. We stored the samples at −20 °C until all study participants completed their evaluations. We used a commercial kit, specifically the QIAamp DNA Micro Kit (Qiagen, Redwood City, CA, USA, Cat. # 56304), to isolate DNA according to the manufacturer’s instructions. We measured DNA concentration and optical density using a spectrophotometer (NP80 All-in-One Spectroscopy, IMPLEN, Munich, Germany). DNA samples with an OD ratio of 1.8 ± 0.1 and a concentration above 40 ng/µL were considered suitable for analysis. The target regions were amplified by polymerase chain reaction (PCR). For both exons, PCR was performed with an initial denaturation at 94 °C for 2 min, followed by 37 cycles of 94 °C for 30 s, 64 °C for 40 s, and 72 °C for 30 s, with a final extension at 72 °C for 3 min. The primer sequences for exon 2 were 5′-GTGGGACAGCTTCATCATTTTG-3′ and 5′-GGGTTCTGTTGCCGAGTC-3′, whereas those for exon 10 were 5′-AGAGCTGTAGGGATGTTG-3′ and 5′-TTCCGTGACTATTGAGTGTG-3′. The PCR products measured 595 base pairs (bp) and 684 bp, respectively. Following a PCR check using gel electrophoresis, we sequenced the PCR products utilizing a commercial kit [Sanger Sequencing Kit (Applied Biosystems, Thermo Fisher Scientific, Waltham, MA, USA, Cat. # A38073)], adhering to the manufacturer’s guidelines, and analyzing them using a capillary electrophoresis instrument (ABI3500, Applied Biosystems, Foster City, CA, USA). We analyzed the results using SeqMan Pro Lasergen Version 12.0 software (DNAStar, Madison, WI, USA). *MEFV* variants were reported according to the reference transcript NM_000243.3. The variants identified or discussed in the present study included c.442G > C (p.Glu148Gln; E148Q), c.2040G > A or c.2040G > C (p.Met680Ile; M680I), c.2080A > G (p.Met694Val; M694V), c.2177T > C (p.Val726Ala; V726A), c.605G > A (p.Arg202Gln; R202Q), c.2230G > T (p.Ala744Ser; A744S), and c.2282G > A (p.Arg761His; R761H). The results were confirmed by bidirectional sequencing.

### 2.3. Statistical Analysis

**Figure 1:**
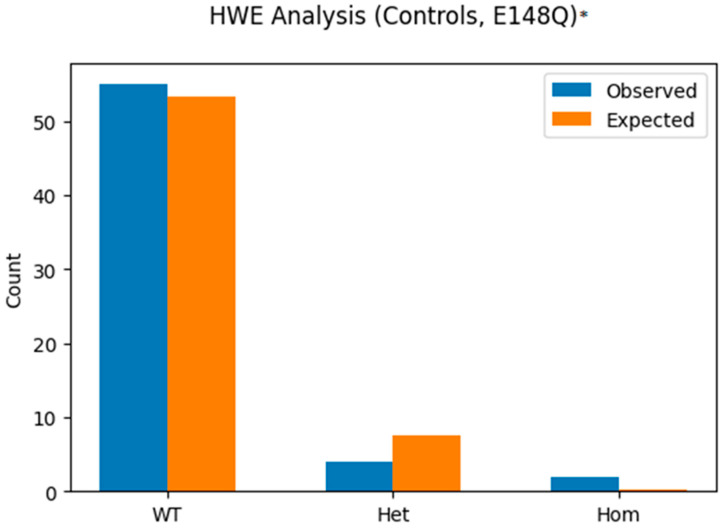
Hardy–Weinberg Equilibrium (HWE) assumptions for control E148Q genotype distribution. * (χ^2^ = 12.18, *p* = 0.0005); Wild type (observed) = 55 and Wild type (expected) = 53.26, Heterozygous (observed) = 4 and Heterozygous (expected) = 7.48, Homozygous (observed) = 2 and Homozygous (expected) = 0.26.

Statistical analyses were performed using SPSS version 15.0 (SPSS Inc., Chicago, IL, USA). Continuous variables, including age, were reported as mean ± standard deviation, and categorical variables were presented as counts and percentages. The Chi-square test was used to compare the distributions of disease and mutation types across other categorical variables (for post hoc analysis, a Bonferroni correction was applied). Differences in mean age between the patient and control groups were evaluated using an independent-samples t-test. In addition, the Hardy–Weinberg equilibrium test (HWE) was used to analyze the distribution of the E148Q genotype (Figure 1). Multivariable logistic regression was used for ARF status, and Fisher’s exact test was used for the association analyses of the main E148Q variant. The relationship between age and disease types or mutations was assessed using Spearman’s rank correlation coefficient. A *p*-value of <0.05 was considered statistically significant in all analyses.

## 3. Results

The study involved 60 patients and 60 healthy controls. Age ranged from 4 to 16 years; the average age was 10.71 ± 3.2 years. The group of patients diagnosed with ARF included 37 females (61.7%) and 23 males (38.3%), whereas the healthy control group included 19 females (31.7%) and 41 males (68.3%).

The results showed that the disease involvement patterns (arthritis, carditis, or arthritis + carditis) differed significantly according to gender among patients diagnosed with ARF (χ^2^ = 14.095, *p* = 0.001). After Bonferroni-adjusted post hoc comparisons, isolated carditis was observed significantly more frequently in female patients (62.2%), whereas combined arthritis and carditis involvement was more common in male patients (56.5%) (Table 1).

Six samples from the patient group failed screening of exon 10 of the *MEFV* gene. In the control group, both exon 2 E148Q and exon 10 analyses failed in 4 samples. There was a significant difference in gender distribution between patients and control groups (χ^2^ = 9.67, *p* < 0.05). The patient group had a greater percentage of females (*n* = 37, 61.7%) than the control group (*n* = 41, 31.7%). The E148Q variant in exon 2 of the *MEFV* gene exhibited a statistically significant difference between the patient and control groups (*p* < 0.05). The E148Q variant was the most prevalent in the patient cohort, identified in 13 samples (Table 2).

Exon 10 mutation analysis indicated no significant differences between the patient and control groups (Table 3). In the patient group, 41 participants (68.3%) demonstrated no mutations in *MEFV* exon 10. We identified M694V as the predominant mutation (*n* = 6), comprising 3 heterozygous, 2 homozygous, and 1 compound heterozygous cases. Additional mutations identified include R202Q, V726A, A744S, M680I, and R761H.

In the control group, 43 participants (76.7%) showed no mutations. The most frequently observed mutations were M694V (*n* = 5; 4 heterozygous plus 1 homozygous) and V726A (*n* = 5). Additional mutations identified include A744S and M680I (see Table 3). We identified a heterozygous mutation in 15 patients, with 10 mutations located in exon 10 and 5 in exon 2. A total of 11 patients exhibited homozygous or compound heterozygous mutations, with 3 in exon 10 and 8 in exon 2 (Table 3). 10 samples (6 from the patient group and 4 from the control group) were discarded because we could not obtain informative results during sequencing.

We identified *MEFV* mutations in 7 ARF patients presenting with carditis, 6 ARF patients demonstrating arthritis, and 10 ARF patients displaying both carditis and arthritis. We identified three mutations in the *MEFV* gene in an 8-year-old male patient with arthritis associated with ARF. In all ARF patient subgroups, E148Q was the most prevalent mutation (Table 4).

As the study included 60 ARF patients and 60 controls, the mean age was 10.85 ± 2.44 years in ARF patients and 10.57 ± 3.87 years in controls. There was a sex imbalance between the groups. ARF patients included 37 females and 23 males, whereas controls included 19 females and 41 males (Table 5).

At the individual level, E148Q was present in 13/60 ARF patients and 6/60 controls. The crude individual-level OR was 2.49 (95% CI 0.88–7.07) and Fisher’s exact *p* = 0.132. At the allele level, E148Q alleles were observed in 21/120 ARF patients and 8/114 controls, based on explicit genotype calls in the dataset. The allele-level OR was 2.81 (95% CI 1.19–6.64) and Fisher’s exact *p* = 0.017 (Table 6).

After adjustment for age and sex in logistic regression, E148Q carrier status had an adjusted OR of 2.90 (95% CI 0.94–9.01; *p* = 0.065). The association was stronger at the allele level, whereas the individual-level adjusted analysis was borderline and not conventionally significant (Table 7 and Table 8).

## 4. Discussion

ARF is an immune-mediated disorder of childhood in which joint and cardiac involvement are the main clinical features. Carditis is particularly important because it accounts for much of the short- and long-term burden of disease and may progress to chronic RHD [17]. In the present series, female patients were more frequent than male patients. Although ARF is generally reported at similar rates in both sexes, RHD has more often been described in females, especially beyond childhood [18,19]. The basis of this difference is not fully understood. It may reflect sex-related differences in immune response, differences in exposure to streptococcal infection, or inequalities in access to prophylaxis [2]. Carditis is often among the earliest manifestations of ARF. The mitral valve is most commonly affected, and mitral regurgitation is the usual valvular finding; aortic regurgitation may accompany it in some patients. The clinical spectrum is broad, ranging from subclinical disease to overt heart failure [20,21]. Previous reports suggest that clinical examination identifies carditis in about 70% of cases, with echocardiography detecting additional subclinical involvement [22]. In our study, clinical carditis was present in 81.6% of patients.

Several autoinflammatory and autoimmune disorders, including rheumatic fever, SLE, and BD, share overlapping clinical features, which can complicate the differential diagnosis. Genetic testing is therefore important both for confirming FMF and for excluding other autoinflammatory conditions with similar presentations [23]. Nearly 80% of FMF cases are attributed to five *MEFV* variants: E148Q, M680I, M694V, M694I, and V726A [9,13,24]. The clinical overlap between ARF and FMF can make the differential diagnosis difficult. Fever, joint symptoms, pericardial involvement, prolonged PR interval, elevated inflammatory markers, and increased anti-streptolysin O titers may be seen in both conditions [25]. *MEFV* variants have also been reported in patients presenting with ARF-like findings [26]. In addition, *MEFV* variation has been associated with several inflammatory disorders, including PFAPA, Behçet’s disease, polyarteritis nodosa, Henoch-Schönlein purpura, systemic-onset juvenile idiopathic arthritis, and adult-onset Still’s disease [10,11,12,27,28,29,30,31,32,33]. Given the frequency of both ARF and FMF in Turkey, it is reasonable to ask whether *MEFV* variation contributes to differences in ARF presentation [5,34].

In our study, the exon 2 E148Q variant was detected more frequently in children with ARF than in controls, across all subgroups. Although the *MEFV* exon 2 E148Q (p.Glu148Gln) variant has long been considered a low-penetrance variant or even a benign polymorphism [24,35,36], recent evidence suggests that it may not be entirely functionally neutral. A recent functional study demonstrated that the E148Q variant can enhance pyrin inflammasome activation, leading to increased ASC speck formation and elevated IL-1β and IL-18 release. The same study also suggested that when E148Q is present in cis with other pathogenic *MEFV* variants, its pro-inflammatory effects may become more pronounced. Therefore, E148Q should not necessarily be interpreted as a highly pathogenic mutation on its own; however, in the presence of a susceptible genetic background or additional inflammatory triggers, it may act as a disease-modifying factor that contributes to dysregulated inflammasome activity. At the same time, current clinical and genetic evidence indicates that the pathogenic significance of E148Q remains controversial, and its interpretation should be made cautiously and always in conjunction with the patient’s clinical findings [37]. Our findings do not resolve this issue, but they support the possibility that E148Q may contribute to susceptibility or phenotypic variation in ARF. Importantly, the observed association between the E148Q variant and ARF remained statistically significant after Bonferroni correction, suggesting that the finding is unlikely to be solely attributable to multiple-comparison bias. However, given the relatively small sample size and the conservative nature of the Bonferroni adjustment, the results should still be interpreted cautiously and validated in larger studies.

In addition, our hypothesis is supported by the broader concept that post-streptococcal immune-mediated diseases may not be determined solely by the infectious trigger, but also by the host’s inflammatory and genetic background. Although ARF and post-streptococcal glomerulonephritis (PSGN) may both follow group A streptococcal infection, only a subset of exposed individuals develop specific clinical phenotypes, suggesting that genetic susceptibility may influence disease expression. Consistent with this view, previous studies have reported a possible association between *MEFV* variants, particularly E148Q, and ARF susceptibility [25,38]. At the same time, other data indicate that *MEFV* mutations may also modify inflammatory phenotypes in rheumatic diseases such as juvenile idiopathic arthritis (JIA) [39]. Moreover, recent functional evidence suggests that E148Q is not necessarily a completely neutral variant, as it may potentiate pyrin inflammasome activation and enhance pro-inflammatory cytokine release [36]. Therefore, similar genetic studies in patients with PSGN and JIA may help clarify whether shared inflammatory triggers result in distinct clinical outcomes via disease-modifying genetic variants.

To contextualize the E148Q heterozygote frequency observed in our cohort, we compared our findings with recent Turkish data published after 2018. In a healthy adult cohort from Havsa, European Turkey, Çakır et al. reported an E148Q allele frequency of 2.6% among 263 unrelated individuals, indicating that E148Q is present at a measurable frequency even in a region with relatively low FMF prevalence [40]. In a larger Turkish cohort analyzed by targeted next-generation sequencing, Kırnaz et al. identified E148Q as one of the most frequent *MEFV* variants, accounting for 17.49% of detected *MEFV* variants among individuals referred for evaluation of autoinflammatory disease [41]. Recent Turkish pediatric studies in non-FMF cohorts have also reported *MEFV* E148Q carriage. In a multicenter study of 597 Turkish children with inflammatory bowel disease, Urgancı et al. found that E148Q was the most common heterozygous *MEFV* variant, particularly in ulcerative colitis and Crohn’s disease groups. Among patients with detected *MEFV* mutations, E148Q accounted for 29.9% of mutation types, and 58 patients were heterozygous for E148Q [42]. Similarly, Ekinci et al. evaluated 144 Turkish children with Henoch–Schönlein purpura without FMF symptoms and identified at least one *MEFV* variant in 50.7% of cases, with E148Q as the most common [43]. More recently, Türkuçar et al. investigated *MEFV* variants in Turkish children with Kawasaki disease and found that 57.9% carried at least one *MEFV* variant allele; however, the most common variant was R202Q rather than E148Q [44]. These findings suggest that E148Q may be encountered in Turkish pediatric patients without classical FMF, especially in inflammatory or vasculitic conditions. Still, these data should not be interpreted as healthy population prevalence.

By contrast, exon 10 variants, mainly M694V and V726A, were identified in 20% of our ARF group, but the difference from controls was not statistically significant. This is noteworthy because exon 10 variants, especially M694V, have more often been linked to severe FMF and AA amyloidosis, whereas V726A is usually associated with a milder phenotype [37,38].

Our results partly parallel those of Tutar et al. [38], who reported a higher frequency of *MEFV* mutations in Turkish patients with RHD. They differ, however, from the adult series reported by Simsek et al. [39]. One possible explanation is the age profile of the cohorts, since ARF is primarily a childhood disease and adult cases may reflect previous or recurrent episodes rather than incident disease [40].

Eleven patients (18.3%) carried heterozygous *MEFV* variants, including E148Q, M694V, M680I, R202Q, V726A, R761H, and A744S. The relatively high carrier frequency observed in Mediterranean populations has often been discussed in terms of heterozygote advantage, namely a more effective inflammatory response against certain pathogens [7,9,25]. If such an advantage exists, the same proinflammatory tendency could also make genetically predisposed individuals more vulnerable to exaggerated sterile inflammation. On that basis, the association we observed with E148Q appears biologically plausible, although it should be interpreted cautiously.

This study indicates that E148Q was more frequently observed in this small ARF cohort, but its clinical significance remains uncertain. Scientific studies conducted by Soylemezoglu (2015) and Kısla Ekinci (2024) on FMF in the Turkish pediatric population also support this [5,45]. These results, along with more and more evidence linking ARF/RHD to FMF, show how important it is to include FMF in the differential diagnosis of ARF/RHD to avoid making the wrong diagnosis.

At the individual genotype level, the E148Q variant was observed in 13 of 60 children with ARF and in 6 of 60 controls in this study. The crude carrier-level association did not reach conventional statistical significance. In allele-level analysis, after adjustment for age and sex, E148Q alleles were more frequent among ARF patients than controls. These findings should be interpreted as preliminary. The E148Q variant was more frequently observed in this small ARF cohort, particularly at the allele level; however, the individual-level-adjusted association was borderline, and the clinical significance of E148Q remains uncertain. The data do not establish causality or prove a specific association with carditis, pericardial effusion, or future rheumatic heart disease. Larger prospective multicenter studies with ancestry control, complete GAS-related clinical data, and longitudinal follow-up are required.

## 5. Limitations

The present study has several limitations. It was conducted at a single center, and the sample size was relatively small, which may have reduced the study’s statistical power. Due to the small cohort size, the study may have been insufficiently powered to detect relatively small effect sizes. At the same time, weaker or more subtle genetic associations could have remained undetected. In addition, the retrospective design may have introduced selection and information bias, and the observed sex imbalance between groups may have influenced the results. Some patients also had incomplete genotyping data, which may have affected the interpretation of variant distribution. Furthermore, potential confounding factors, including environmental, socioeconomic, and additional genetic variables, could not be fully adjusted for in the analyses. Long-term follow-up data on the development of rheumatic heart disease and other late complications were unavailable, limiting the ability to assess the prognostic significance of the identified variants. Functional analyses were also beyond the scope of the current study. In addition, echocardiographic assessments were not evaluated in a genotype-blinded manner, which may have introduced observational bias; in addition, the exclusion of variables such as ASO/anti-DNase B, ESR/CRP, region/ethnicity, kinship, colchicine use, and long-term follow-up from the study should be considered a limitation. Therefore, the findings should be interpreted with caution, and the data should be carefully evaluated, particularly when considering the potential clinical significance of the identified variants.

Furthermore, this study should be considered exploratory and requires validation in larger, independent, and preferably multicenter cohorts. It should also be emphasized that an observed genetic association does not necessarily imply causation, and that most patients with ARF do not carry *MEFV* variants. In addition, the pathogenic significance of the E148Q variant remains controversial, as it has been variably classified as a low-penetrance mutation, a disease-modifying variant, or a benign polymorphism in different studies. Larger studies with molecular and longitudinal follow-up are needed to clarify whether these variants play a direct role in the ARF phenotype or instead reflect a broader inflammatory predisposition.

## 6. Conclusions

The findings of this study may suggest a possible association between pericardial effusion and carditis in patients with acute rheumatic fever and the *MEFV* exon 2 E148Q variant. Significant results were obtained at the E148Q allele level; however, in the adjusted analysis at the individual level, the results were borderline. Consequently, larger, preferably multicenter studies with molecular and longitudinal follow-up are needed to determine whether these variants play a direct role in the ARF phenotype or instead reflect a broader inflammatory predisposition.

## Figures and Tables

**Table 1:** The distribution of clinical involvement patterns (arthritis, carditis, and combined arthritis-carditis) among patients diagnosed with acute rheumatic fever (ARF).

Gender	Patients with ARF			*χ*^2^: 14.095 *p* = 0.001
	Arthritis n (%)	Carditis n (%)	Arthritis + Carditis n (%)	Total n (%)
Female	4 (10.8) ^a^	23 (62.2) ^b^	10 (27) ^a^	37 (61.7)
Male	7 (30.4) ^a^	3 (13) ^b^	13 (56.5) ^a^	23 (38.3)
Total	11 (18.3)	26 (43.3)	23 (38.3)	60 (100)

χ^2^: Chi-square. Rows with different superscript letters (^a, b^) indicate statistically significant differences in proportions between groups according to the Bonferroni-adjusted comparisons (*p* < 0.05).

**Table 2:** Distribution of the *MEFV* Exon 2 E148Q variant in Patient and Control Groups.

	Groups		*χ*^2^ = 5.648 *p* = 0.017
E148Q Variant	Patient # of Alleles (%)	Control # of Alleles (%)	Total # of E148Q Alleles (%)
Present	21 (72.4) ^a^	8 (27.6) ^b^	29 (100)
Absent	99 (48.7) ^a^	104 (52.3) ^b^	203 (100)
Total	120	112	232

χ^2^: Chi-square. Rows with different superscript letters (^a, b^) indicate statistically significant differences in proportions between groups according to the Bonferroni-adjusted comparisons (*p* < 0.05). #: Number.

**Table 3:** Distribution of the *MEFV* Exon 10 Mutation in Patient and Control Groups.

Exon 10 Mutations	Groups		*χ*^2^: 6.490 *p* = 0.593
	Patients, *n* (%)	Control, n (%)	Total (*n*)
Absent	41 (75.9)	43 (76.7)	84
R202Q (+/−)	1 (1.85)	0	1
M694V (+/−)	3 (5.55)	4 (7.14)	7
M694V (+/+)	2 (3.7)	1 (1.78)	3
V726A (+/−)	1 (1.85)	5 (8.92)	6
M694V/M680I	1 (1.85)	0	1
A744S (+/−)	2 (3.7)	1 (1.78)	3
M680I (+/−)	2 (3.7)	2 (3.57)	4
R761H (+/−)	1 (1.85)	0	1
Total	54 (100)	56 (100)	110

(+/−): Heterozygous; (+/+): Homozygous; M694V/M680I: compound heterozygous;. *χ*^2^: Chi-square.

**Table 4:** Demographic Characteristics and *MEFV* Gene Mutation Distribution in ARF Patients with Arthritis, Carditis, and Combined Arthritis-Carditis.

	*MEFV* Gene Mutations	Age	Gender	Heart Valve Lesion
ARF patients with carditis	E148Q (+/+)	10	Female	1° MR + 1° AR
	E148Q (+/+)	14	Female	1° AR + Mild TR
	E148Q (+/−)	12	Female	2° MR + Mild TR
	M694V (+/+)	8	Male	1° MR + trace PR
	M694V (+/+)	12	Female	1° MR
	R202Q (+/−)	8	Female	2° MR
	R761H (+/−)	15	Female	1° AR + Mild TR + trace PR
ARF patients with arthritis	E148Q/E148Q/M694V	8	Male	N/A
	E148Q (+/+)	12	Male	N/A
	E148Q (+/−)	12	Female	N/A
	E148Q/M694V	12	Male	N/A
	M694V/M680I	11	Male	N/A
	M680I (+/−)	9	Male	N/A
ARF patients with carditis plus arthritis	E148Q (+/+)	6	Female	3° MR + 1°AR + Mild TR + pericardial effusion
	E148Q (+/+)	15	Male	3° MR + 2° AR + trace PR
	E148Q (+/+)	12	Female	1° MR
	E148Q (+/+)	12	Female	1° MR + 1° AR + trace PR
	E148Q (+/−)	12	Male	1° MR
	E148Q (+/−)	10	Male	1° MR
	A744S (+/−)	13	Male	1° MR + trace PR
	A744S (+/−)	8	Male	1° MR
	V726A (+/−)	12	Female	1° MR + Mild TR
	M680I (+/−)	10	Female	1° MR + 1° AR

(+/−): Heterozygous; (+/+): Homozygous; MR: mitral regurgitation; TR: Tricuspid regurgitation; PR: Pulmonary regurgitation; AR: Aortic regurgitation; N/A: Not applicable.

**Table 5:** E148Q genotype summary among the groups.

Variable	ARF Patients	Controls
*n*	60	60
Age, mean ± SD	10.85 ± 2.44	10.57 ± 3.87
Female/Male	37/23	19/41
E148Q wild-type	47	51
E148Q heterozygous	5	4
E148Q homozygous	8	2
Non-informative	0	3

**Table 6:** The main E148Q association analysis.

Analysis	ARF	Control	OR	95% CI	*p*-Value *
Individual-level carrier analysis	13/60	6/60	2.49	0.88–7.07	0.132
Allele-level analysis	21/120	8/114	2.81	1.19–6.64	0.017

* *p*-values were obtained from Fisher’s exact test.

**Table 7:** Multivariable logistic regression for ARF status.

Variable	Adjusted OR	95% CI	*p*-Value
E148Q carrier	2.90	0.94–9.01	0.065
Age	0.98	0.86–1.11	0.717
Female sex	3.83	1.73–8.50	0.001

**Table 8:** ARF phenotype by E148Q carrier status.

E148Q Status	Total ARF Patients	Carditis Only	Arthritis Only	Arthritis + Carditis
E148Q (−)	47	23	7	17
E148Q (+)	13	3	4	6

(−): absent; (+): present.

## Data Availability

The datasets generated and analyzed in this study are available from the corresponding author upon reasonable request.

## Abbreviations

The following abbreviations are used in this manuscript:

ARF	Acute Rheumatic Fever
GAS	Group A β-haemolytic streptococci
FMF	Familial Mediterranean Fever
RHD	Rheumatic Heart Disease
*MEFV*	Mediterranean fever gene
HSP	Henoch-Schönlein Purpura
SLE	Systemic Lupus Erythematosus
BD	Behçet’s Disease
PN	Polyarteritis nodosa
WHO	World Health Organization
ECG	Electrocardiogram
ECHO	Echocardiogram
PCR	Polymerase Chain Reaction
SPSS	Statistical Package for the Social Sciences
